# High incidence of leptospirosis in an observational study of hospital outpatients in Vanuatu highlights the need for improved awareness and diagnostic capacities

**DOI:** 10.1371/journal.pntd.0006564

**Published:** 2018-06-04

**Authors:** Junior George Pakoa, Marie-Estelle Soupé-Gilbert, Dominique Girault, Dexter Takau, Justina Gaviga, Ann-Claire Gourinat, Arnaud Tarantola, Cyrille Goarant

**Affiliations:** 1 Department of Pathology and Medical Diagnostic Laboratory Medicine PMB 9013, Vila Central Hospital, Ministry of Health, Port Vila, Vanuatu; 2 Institut Pasteur International Network, Institut Pasteur in New Caledonia, Leptospirosis Research and Expertise Unit, Nouméa Cedex, New Caledonia; 3 Institut Pasteur International Network, Institut Pasteur in New Caledonia, Serology and Molecular Diagnostics Unit, Nouméa Cedex, New Caledonia; 4 Centre Hospitalier Territorial de Nouvelle-Calédonie, Medical Biology Laboratory, Nouméa Cedex, New Caledonia; 5 Institut Pasteur International Network, Institut Pasteur in New Caledonia, Medical Epidemiology Research Unit, Nouméa Cedex, New Caledonia; University of Minnesota, UNITED STATES

## Abstract

**Background:**

Estimates of leptospirosis morbidity identified Oceania as the region with highest burden. Besides Australia and New Zealand, Oceania is home of Pacific Island Countries and Territories, most of which are developing countries facing a number of challenges. Their archipelago geography notably affects health infrastructure and access to healthcare. Although human leptospirosis was formerly identified in Vanuatu, there is a lack of knowledge of this disease in the country. We aimed to identify leptospirosis in outpatients visiting the hospital.

**Methodology/Principal findings:**

We conducted a clinical study to investigate leptospirosis as a cause of non-malarial acute febrile illness in Vanuatu. A total 161 outpatients visiting the outpatient clinics at Port Vila Central Hospital for internal medicine were recruited over 20 month. We showed that leptospirosis significantly affects humans in Vanuatu: 12 cases were confirmed by real-time PCR on acute blood samples (n = 5) or by high serology titers evidencing a recent infection (MAT titer ≥800 or ELISA≥18 Units, n = 7). A high rate of positive serology was also evidenced, by MAT (100<titer<800, 9 patients) or ELISA IgM (ELISA≥12 Units, 20 patients, including 6 also positive in MAT), showing frequent exposure to pathogenic leptospires, notably from serogroup Australis.

**Conclusions/Significance:**

The high numbers of both seropositive patients and acute leptospirosis cases observed in outpatients visiting Port Vila Central Hospital suggest a high exposure to pathogenic *Leptospira* in the population studied. The MAT serology pointing to serogroup Australis as well as exposure history suggest that livestock animals largely contribute to the burden of human leptospirosis in Vanuatu. The analysis of residential and travel data suggests that the risk might even be higher in other islands of the Vanuatu archipelago. Altogether, our study emphasizes the need to increase awareness and build laboratory capacity to improve the medical care of leptospirosis in Vanuatu.

## Author summary

Leptospirosis is thought to impose its highest burden to tropical island populations, especially in the Pacific region of Oceania. Yet, very few information has ever been reported from some of the Pacific Island Countries and Territories, including Vanuatu. In this study, we aimed at evidencing leptospirosis in a convenience sample of outpatients visiting Port Vila Central Hospital, the major hospital of this archipelago country in the South Pacific. Using reference laboratory techniques, our study confirms a significant number of acute leptospirosis cases, as well as a high level of previous exposure in outpatients visiting this hospital. Risk factor analysis point to livestock and lifestyle as important factors exposing to leptospirosis in this developing country. Furthermore, there is evidence that the disease probably has a higher incidence in remote islands outside of the capital of Efate in this very wide archipelago. Taken together, our results point to a need of increased awareness, notably in peripheral health centers and of better access to laboratory confirmation for surveillance.

## Introduction

Leptospirosis is among the most widespread zoonosis worldwide. Pathogenic leptospires colonize the renal tubules of asymptomatic chronically infected reservoir mammals, including rodents and livestock. The bacteria are then shed through the urine in the environment, where they can survive for weeks to months in favorable hot and humid hydro-telluric environments. This epidemiological trait is regarded as the main cause of leptospirosis seasonality, with highest incidence being observed during hot and rainy periods globally. Most human infections occur in freshwater or mud, during occupational (notably agriculture and farming) or recreational (e.g. freshwater bathing) activities [1]. Its global morbidity and mortality were recently estimated to 1.03 million cases and 58,900 deaths annually, mostly in resource-poor countries [2]. The burden imposed to populations globally is in the same range as the burden of Leishmaniosis, Schistosomiasis or Lymphatic filariasis [3]. The death toll of leptospirosis is therefore five times the total number of fatalities of the 2013–2016 Ebola outbreak every year [4]. Interestingly, both estimates of the burden [2,3] place Oceania as the region of highest incidence and highest burden by far. Yet, very little is known on human leptospirosis in many Pacific Island Countries and Territories (PICTs), earning it the well-deserved name of neglected tropical disease.

**Fig 1:**
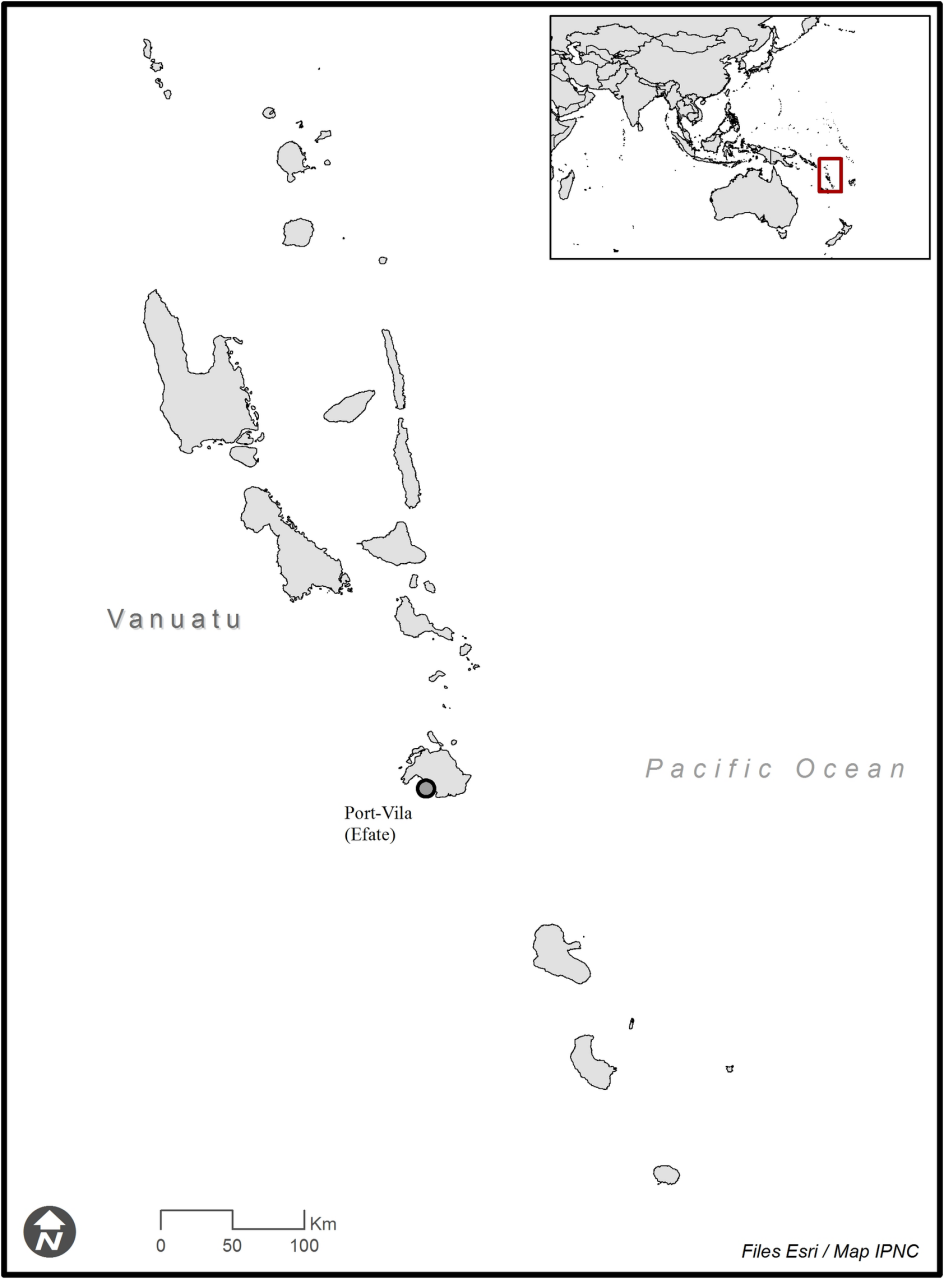
Map showing the Vanuatu archipelago and the capital Port Vila.

Many of these countries including Vanuatu experience climatic and environmental conditions prone to favor leptospirosis. Furthermore, some population groups experience precarious living and sanitation conditions. It is likely that human leptospirosis occurs at high rates in these settings, a risk that might also increase because of climate change. Vanuatu is an island nation located in the South Pacific. This archipelago of volcanic origin is made of more than 80 islands (with 65 being inhabited) spread over ca. 1,300 km from North to South (Fig 1). Vanuatu has a ca. 272,500 population, predominantly rural. The rural lifestyle involves frequent exposure to natural freshwater bodies, subsistence agriculture and farming, including free-ranging pigs, which are also used in customs ceremonies. Vanuatu was also given the highest risk of exposure to natural disaster worldwide, notably being highly exposed to earthquakes and tropical cyclones [5]. In Vanuatu, leptospirosis was first identified in cattle through a seroprevalence study in the 1980s [6]. The first reported cases of human leptospirosis were identified in patients in Port Vila by the New Caledonian Institut Pasteur in the early 1990s [7]. An additional eight cases were reported from Santo Island in the early 2000s [8]. A published case of an Australian tourist contracting (ultimately fatal) leptospirosis during a holiday in Vanuatu in the early 2000’s highlights the leptospirosis risk associated with freshwater bathing [9]. Lastly, a regional study in 2003–2005 included 10 patients from Vanuatu, showing positive serology in one case [10]. Taken together, published information on human leptospirosis in Vanuatu is scant. When considering domestic mammals (as both possible susceptible hosts and reservoirs for human infections), farmers proved to have a limited knowledge of the disease, although leptospirosis was ranked first in a regional priority list for farm animal biosecurity established by a panel of regional experts from the animal health and production sectors [11].

In this study, we aimed at quantifying the number of leptospirosis cases and the seroprevalence of anti-Leptospira antibodies in patients visiting the outpatient clinic of the internal medicine ward at Vila Central Hospital, the national reference hospital in Vanuatu.

## Materials and methods

### Ethics statement

Written informed consent was obtained from all included patients or their legal representative for minors. The study was endorsed by Institut Pasteur under number RBM 2012.33 after approval by Comité de Protection des Personnes Ile de France and was approved by the Comité Consultatif sur le Traitement de l’Information en matière de Recherche dans le domaine de la Santé (CCTIRS) with number 12.770 after validation by Vanuatu Ministry of Health. The anonymous database was registered with the French Commission Nationale de l’Informatique et des Libertés (CNIL). The Strobe checklist is presented as S2 Supporting information file.

### Setting

Port Vila Central Hospital (VCH) is the 150-bed central hospital of Vanuatu. It is located in Port Vila, the capital of Vanuatu on the island of Efate. The Efate population is ca. 41,600 inhabitants, including ca. 25,000 in Port Vila. Inter-island migration in Vanuatu, however, is intense and patients from other islands and provinces sometimes refer to VCH. The outpatient clinics receives an average of 1,400 patients per week.

### Inclusion criteria, patients, samples and data collection

The aim of the study was to identify leptospirosis cases by investigating patients presenting at the outpatient clinic at VCH in Vanuatu. We therefore used the leptospirosis clinical description by the World Health Organization [12]. The following inclusion criteria were selected: acute febrile illness with headache and myalgia and any of the following: prostration, conjunctival injection, meningeal syndrome, anuria or oliguria and/or proteinuria, icterus, hemorrhages, Cardiac arrhythmia or deficiency, skin rash (S1 Supporting Information). However, these inclusion criteria were not used in routine practice and no rigorous inclusion criteria were actually used.

A sample of patients aged 15 year old or older visiting the outpatient clinic at Vila Central Hospital (VCH), Port Vila, Vanuatu were included in the study. After medical evaluation addressing the patient complaints, the medical practitioner decided whether the clinical presentation was compatible with leptospirosis. If so, the consent of the patient or his/her legal representative was sought after information on the disease and on the goals of the study. After having an informed consent form signed, the patients were referred to the laboratory where they were screened for malaria. Only malaria-negative patients were included in this study. The medical staff in the laboratory was trained about leptospirosis, about the process of the study and the administration of a standardized questionnaire about patient exposure risk factors (occupation and contact with various water sources and with animals in the three weeks before onset of symptoms). This questionnaire was administered in local pidgin language (bislama) and written in either English or French (S1 Supporting information). Demographic data (sex, age), clinical presentation and the data about possible exposure were transferred to Institut Pasteur in New Caledonia (IPNC) after de-identification (S1 Supporting information).

### Biological analyses and case definitions

In VCH laboratory, patients were first screened for malaria by the classical thick blood smear technique from capillary blood. Blood was then collected in a plain tube and serum was used for a malaria rapid diagnostic test (CareStart Malaria HRP2/pLDH(pf/PAN)Combo). When negative, a *Leptospira* serology was made using Leptospira IgM ELISA (Panbio). In some patients, urine was also collected and immediately frozen. The remaining serum and the urine when available were sent together with the case documentation form to IPNC. At IPNC, DNA was extracted from 200 μL serum or urine using the MagnaPure LightCycler (Roche Diagnostics). *Leptospira* DNA was detected by real-time PCR targeting the *lipL32* gene, specific of pathogenic leptospires [13], on a LightCycler 480. The microscopic agglutination test (MAT)—the reference technique for *Leptospira* serology—was also used (when sufficient volume was available) using a regional 24-strain panel described before [10] and as recommended by WHO. Positive serological cutoff were set at a titer of 100 for MAT or 12 for ELISA [14]. Probable acute leptospirosis cases were defined as patients with a high serological titer (MAT ≥ 800 or ELISA ≥ 18), suggestive of a recent infection. The serogroup with the highest titer was considered as the putative infecting serogroup. Confirmed acute leptospirosis cases were defined as patients with a positive qPCR.

### Statistical analyses

Data were entered in an Excel spreadsheet (Microsoft Corporation, Redmond, WA, USA) and analyzed using Stata 13 (College Station, TX, USA). Variables were analyzed descriptively before bivariate analysis explored the association between outcome (leptospiral disease and then positive serology) with exposure variables (sociodemographic, clinical, environmental exposures) using a 5% statistical threshold. Categorical variables were dichotomized and successively tested using bilateral Fisher’s tests. Variables associated with a significance level of 0.2 were integrated into a logistic regression model to compute odds-ratios.

## Results

### Patient statistics

From January 2013 to August 2014, 161 patients (84 females and 77 males, M:F = 0.92) were included. Patients’ age ranged from 15 to 75 years (mean 34; median 30; IQR 23–40). Most patients included in our series visited the outpatient clinic during the first quarter of 2013 (n = 45) or 2014 (n = 43) and 128 (79.5%) patients visited during the first half of both years taken together. The quarterly distribution of the samples are summarized in Table 1. The chief complaint was “feeling unwell”, with headache (79%) and myalgia/arthralgia (75%) being most frequently reported (Table 2).

**Table 1:** Distribution of patient gender, serostatus and clinical status, by quarter, Vanuatu 2013–2014.

	2013				2014			Total
	1^st^ quarter#	2^nd^ quarter	3^rd^ quarter	4^th^ quarter	1^st^ quarter	2^nd^ quarter	3^rd^ quarter	
Females	22	11	10	1	25	11	4	84
Males	23	6	10	0	18	12	8	77
Positive serology	11	4	6	0	5	2	1	29
*Percentage positive*	*24*.*4%*	*23*.*5%*	*30%*	*0%*	*11*.*6%*	*8*.*7%*	*8*.*3%*	*18%*
Cases	7	0	3	0	1	0	1	12
Total patients	45	17	20	1	43	23	12	161

# 1^st^ quarter: January to March; 2^nd^ quarter: April to June; 3^rd^ quarter: July to September; 4^th^ quarter: October to December. The hot and rainy season peaks during the first quarter (January to March).

**Table 2:** Summary of demographics and complaints in the patient population.

Age class (years)	Females (n = 84)	Males (n = 77)	Total
[15–20]	15	11	26
[21–30]	34	23	57
[31–40]	20	20	40
[41–50]	7	12	19
[51–60]	4	6	10
[61–75]	3	5	8
ND	1		1
Complaints			
Headache	86.1%	77.6%	155
Myalgia / arthralgia	79.2%	77.6%	153
Fever or chills	44%	41.6%	161
Vertige / malaise	28.6%	26%	161
Digestive symptoms	3.6%	2.6%	161

All patients but two (unknown signs and symptoms) reported or presented at least one of the following: Headache (n = 127; 79.9%); fever or chills (n = 69; 43.4%); myalgia or arthralgia (n = 120; 75.5%); prostration (n = 27; 17%); respiratory discomfort or cough (n = 17; 10.7%); jaundice (n = 11; 6.9%); oliguria or anuria (n = 9; 5.7%). However, only 23 patients (14.3%) fulfilled the inclusion criteria initially defined for the study.

### Laboratory analyses

All blood samples were tested for leptospiraemia using qPCR. In addition, 53 urine samples from the same patients were also tested. No urine sample was positive, but five blood samples were qPCR-positive. For serology and following logistical issues, 31 samples were tested by IgM ELISA only, 45 by MAT only and 85 with both techniques. These results are summarized in Table 3.

**Table 3:** Biological diagnostic techniques for leptospirosis used in this study.

	*Leptospira* qPCR		*Leptospira* serology		
	Blood only	Blood & Urine	ELISA only	MAT only	ELISA & MAT
Number of patients	108	53	31	45	85
Total	161		161		

### Acute leptospirosis cases and risk factors

Twelve (7.45%) cases of acute leptospirosis were identified as confirmed (n = 5) or probable (n = 7). Only two of these cases were reported to fulfill the inclusion criteria. The seasonal distribution of samples was biased towards the rainy season during the first quarter of each year (Table 1) and was too irregular to document a possible seasonal pattern. Exposures significantly associated with clinical leptospirosis in bivariate analysis were male gender, the use of water from a well or a natural source at home, fishing in freshwater or contact with pigs. Residing at least part-time on islands other than Efate also was associated with clinical leptospirosis. Detailed statistics and the corresponding Odds Ratio are presented in Table 4. A multilevel logistic regression found that only living at least part time on another island than Efate (OR 8.64; CI95% 1.22–61.41; p = 0.03) remained significantly associated with acute leptospirosis after adjustment for other factors. From a clinical standpoint, cough or hemoptysis were significantly associated with acute leptospirosis (OR = 9.9 CI95% [1.4994; 75.4296]; p = 0.008).

**Table 4:** Details of the analysis of risk factors for acute leptospirosis and leptospirosis positive serology.

		Clinical leptospirosis				Positive leptospirosis serology			
	Total (n)	Cases (n)	% cases	Unadjusted OR [CI95%]; p	Adjusted OR [CI95%]; p	Seropositives (n)	% seropositive	Unadjusted OR [CI95%]; p	Adjusted OR [CI95%]; p
**Sex**									
Male	77	10	13.0%	6.12 [1.30–28.89]; 0.02	3.98 [0.63–25.27]; 0.14	17	22.1%	1.7 [0.75–3.84]; 0.2	0.69 [0.21–2.26]; 0.54
Female	84	2	2.4%	Ref.	Ref.	12	14.3%	Ref.	Ref.
**Water at home**									
From a well or a source	21	5	23.8%	6.20 [1.69–22.66]; 0.006	1.38 [0.20–9.73]; 0.75	8	38.1%	4.19 [1.5–11.69]; 0.006	2.05 [0.46–9.17]; 0.34
Other origin of water	125	6	4.8%	Ref.	Ref.	16	12.8%	Ref.	Ref.
**Freshwater fishing**									
Fishing in freshwater	19	4	21.1%	4.53 [1.18–17.32]; 0.03	9.67 [0.73–127.42]; 0.08	6	31.6%	2.77 [0.93–8.23]; 0.07	0.73 [0.14–3.89]; 0.71
No fishing in freshwater	126	7	5.6%	Ref.	Ref.	18	14.3%	Ref.	Ref.
**Contact with surface water**									
Contact	31	4	12.9%	2.72 [0.72–10.32]; 0.14	0.89 [0.15–5.45]; 0.90	11	35.5%	4.36 [1.71–11.10]; 0.002	2.76 [0.83–9.23]; 0.10
No contact	116	6	5.2%	Ref.	Ref.	13	11.2%	Ref.	Ref.
**Hunting**									
Hunting	8	2	25.0%	5.12 [0.89–29.58]; 0.07	1.00 [0.77–13.21]; 1.0	1	12.5%	0.71 [0.08–6.04]; 0.75	-
No hunting	131	8	6.1%	Ref.	Ref.	22	16.8%	Ref.	-
**Contact with animals**									
Contact with pigs	44	6	13.6%	4.22 [1.13–15.78]; 0.03	2.71 [0.54–13.64]; 0.23	14	31.8%	3.52 [1.49–8.30]; 0.004	2.68 [0.87–8.27]; 0.09
No contact with pigs	111	4	3.6%	Ref.	Ref.	13	11.7%	Ref.	Ref.
Contact with dogs or cats	148	8	5.4%	0.23 [0.04–1.26]; 0.09	0.21 [0.2–2.10]; 0.18	24	16.2%	0.45 [0.11–1.87]; 0.27	-
No contact with dog or cat	10	2	20.0%	Ref.	Ref.	3	30.0%	Ref.	-
Contact with horses, goats or sheep	9	0	0.0%	NE	-	3	33.3%	2.86 [0.66–12.32]; 0.159	-
No contact with horses, goats or sheep	141	8	5.7%	-	-	21	14.9%	Ref.	-
Contact with cattle	13	2	15.4%	2.73 [0.51–14.46]; 0.24	-	6	46.2%	5.60 [1.68–18.65]; 0.005	2.37 [0.48–11.70]; 0.30
No contact with cattle	128	8	6.3%	Ref.	-	17	13.3%	Ref.	Ref.
**Rodents seen in the vicinity**									
Rodents seen	142	11	7.7%	NE	-	25	17.6%	0.85 [0.17–4.25]; 0.85	-
No rodents seen	10	0	0.0%	-	-	2	20.0%	Ref.	-
**Island of residence**									
At least part-time on an island other than Efate	47	9	19.1%	8.76 [2.25–34.06]; 0.002	**8.64 [1.22–61.41]; 0.03**	13	27.7%	2.34 [1.02–5.37]; 0.04	1.59 [0.51–4.99]; 0.43
Living full time in Efate only	114	3	2.6%	Ref.	Ref.	16	14.0%	Ref.	Ref.
**Occupational risk**									
Agriculture, gardening, farming, building, butcher	28	3	10.7%	1.50 [0.35–6.44]; 0.59	-	8	28.6%	1.51 [0.57–4.01]; 0.41	-
No known occupational risk	81	6	7.4%	Ref.	-	17	21.0%	Ref.	-

OR: odds-ratio; CI95%: 95% confidence interval; NE: Not estimable/not convergent

### *Leptospira* seropositivity and risk factors

A positive serology was identified in 29 (18%) patients (including nine of the 12 cases). Positive serology was more frequent in males than females, but this difference did not reach statistical significance (p = 0.22). Patients residing at least part time on another island than Efate had a slightly and statistically borderline risk of having a positive serology compared to patients residing on Efate (27.7% vs. 14.3%, p = 0.07). Contact with freshwater (in rivers, lakes or irrigated culture) was a risk factor (p = 0.004), as was using water from a well or natural source (p = 0.008). Lastly, contact with pigs (p<0.01) or with cattle (p<0.01) were statistically significantly associated with seropositivity in bivariate analysis. A logistic regression model integrating these various factors found no significant risk factor, only contact with pigs retained borderline significance after adjustment for all other factors. Detailed statistics and Odds Ratios are presented in Table 4.

Using MAT, 130 samples were tested, yielding 15 (11.5%) positive results. In our series, a higher titer was found for serogroups Pyrogenes (n = 1), Icterohaemorrhagiae (n = 1), Louisiana (n = 1) and Panama (n = 1), three had co-agglutinations with no serogroup giving a higher titer, and serogroup Australis was the most prevalent, with eight positive sera.

## Discussion

This study was aimed to identify leptospirosis cases in patients visiting the outpatient clinic at Port Vila Central Hospital in Vanuatu. The patient selection used has a number of biases. The exclusion of malaria cases had a very low impact on the patient selection, since malaria is considered eradicated in the Shefa province and very few malaria cases had to be excluded. Most patients were also recruited during the hot and rainy season. Because a strong seasonality is frequently reported in leptospirosis incidence, including in the neighboring archipelagoes of New Caledonia and Wallis & Futuna [14,15], this bias is possibly leading to an over-estimation of the incidence of the disease annually. The patient recruitment initially aimed at including patients with a clinical presentation recognized as a standard presentation in leptospirosis by WHO [12]. However, the inclusion criteria were not strictly respected and only 14.3% of patients fulfilled this suspicion definition and less than half of the patients were febrile or reported previous fever or chills. However, even if patients were not selected using a rigorous leptospirosis clinical suspicion, they were selected for a leptospirosis suspicion by medical doctors. This suggests that more leptospirosis cases would have been identified if the selection had focused strictly on acute febrile patients, but also that the number of leptospirosis is higher than in the total outpatient population. Taken together, the patient selection used in this study prevents from using the results of our study to evaluate the burden of leptospirosis in the country.

Still, we evidenced both a significant number of incident leptospirosis cases and a high seroprevalence for *Leptospira* among outpatients. Yet, there is currently no specific health policy and very poor awareness on this disease in the country. Our results highlight the need to strengthen awareness of the medical community and reinforce laboratory diagnostic capabilities.

Recent evaluations of the leptospirosis burden globally have identified Oceania as the region of highest morbidity [2] and with the highest burden in terms of Disability Adjusted Life Years [3]. Leptospirosis is also considered the infectious disease posing the greatest risk for livestock health and productivity [11]. In Vanuatu, however, only two studies have investigated leptospirosis in humans [7,8]. Despite evidence of leptospirosis in returning tourists after exposure to freshwater [9], leptospirosis is most frequently not considered for routine patient diagnostics in Vanuatu. Here, we used a sample of patients who visited the outpatient clinic of Port Vila Central Hospital. Although only a minority of patients had evidence of fever or reported fever or chills (43%), all clinically documented patients had at least one clinical sign compatible with leptospirosis. Our patient study likely does not reflect VCH outpatients, but patients were not strictly selected for suspected leptospirosis. Using post-hoc analysis with rigorous diagnosis criteria and case definitions, we were able to identify 12 (7.45%) leptospirosis cases. Because the patient sample of this study is season-biased with a majority (80%) of inclusions in the first half of the year (encompassing the hot rainy season), this incidence cannot be extrapolated to the entire year. It can nevertheless be assumed that this number reflects a high incidence of leptospirosis among outpatients visiting Port Vila Central Hospital, at least during the hot rainy season.

Combining MAT and ELISA results, we evidenced 18.0% serological positivity. The seroprevalence observed was 11.5% using the reference technique MAT, a technique known to be poorly sensitive at the early stage of the disease, possibly leading to false negative results in early samples [16]. In contrast, it was 18.1% using ELISA, a technique allowing earlier detection of antibodies but with lower specificity compared to MAT, therefore possibly leading to some level of false positivity [17]. The risk factors associated with a positive serology point to well-known risks, such as freshwater exposure and contact with farm animals, notably pigs and cattle, but not goats, horses or sheep. Exposure to freshwater during fishing activities was also a risk for leptospirosis. Interestingly, MAT results point to Australis as the dominant serogroup, which is frequently involved in human cases in various Pacific Islands and most frequently pointing to a swine reservoir [10,14,18–22]. This finding together with the fact that contact with pigs was a significant risk factor for overt disease and borderline for positive serology points to pigs as a potential contributor to human leptospirosis in Vanuatu, as observed in other PICTs. Another finding was that patients living at least part time in another island in the archipelago also had a higher risk of presenting clinical leptospirosis. Though this might be caused by a recruitment bias of patients forwarded by peripheral health centers, this also suggests that leptospirosis incidence could be yet higher in other islands of Vanuatu, where medical care is frequently harder to access. This may be explained by a different, more rural lifestyle, in which farming, agriculture and freshwater exposure are part of everyday activity for most of the population. Because severe leptospirosis fatality is high in the absence of intensive care and renal replacement therapies, this finding suggests that the hidden morbidity of leptospirosis is possibly associated with a high mortality on these islands.

One major cause for insufficient recognition of this disease in the region relates to the diagnostic challenges. Because the clinical presentation of leptospirosis is non-specific and polymorphic, reliable diagnosis involves detecting leptospires in biological fluids (mostly blood or urine) or by seroconversion (requiring two successive samples) as detected by the micro-agglutination test (MAT) [23]. These diagnostic techniques should be introduced in Vanuatu with scientific and technical support from reference centers with relevant experience. Reliable early diagnosis paves the way for effective case management, greatly improves patient prognosis and provides information for public health policymaking. For public health managers, the acknowledgement that this zoonotic and environmental disease is present and an assessment of its burden can help guide decisions affecting local or regional health priorities, implementation of veterinary public health policy, physical planning (access to clean water sources or waste management) or the control of wild or stray animals. Further research is needed to gain a precise knowledge of the burden of leptospirosis in this archipelago, including in peripheral health centers. This could be achieved by the routine implementation of real-time PCR and IgM ELISA together with the support of a reference center for MAT [23]. Because this study suggests a significant role of non-rodent reservoir animals as a source of human leptospirosis, the role of livestock should also be investigated.

To conclude, our exploratory study shows that a significant number of patients visiting the outpatient clinic at Port Vila Central Hospital have leptospirosis, highlighting the need for increased awareness in the medical community as well as in the general population, especially during the hot and rainy season from January to April. Because the diagnosis of leptospirosis requires biological confirmation, medical laboratories should also be trained and supported for this purpose.

## Supporting information

S1 Supporting information Patient documentation form. (PDF)

S2 Supporting information Strobe checklist. (PDF)
