# Supplementary material for: High incidence of leptospirosis in an observational study of hospital outpatients in Vanuatu highlights the need for improved awareness and diagnostic capacities
Source: PLoS Negl Trop Dis. 2018 Jun 4;12(6):e0006564. doi: 10.1371/journal.pntd.0006564 (PMC6002134; doi:10.1371/journal.pntd.0006564)
Supplement: S1 Supporting information — (PDF) [file pntd.0006564.s001.pdf]

Leptospirosis suspicion in hospitalized patients

~~**Inclusion criteria**: Acute febrile syndrome with headaches and myalgia PLUS AT LEAST one of the following: prostration – conjonctival injection – meningeal syndrome – anuria or oliguria and/or proteinuria – icterus – haemorrages – Cardiac arythmia – cardiac deficiency – skin rash~~

Place of recruitment Hospital ____________________ Service ____________________

Patient Hospitalisation date |__|__| |__|__| |__|__|__|__| **Anonymous code |__|__||__|__|__|__|**

Family name ____________________ Given name ____________________

City / Island of residence ____

Date of birth |__|__| |__|__| |__|__|__|__| Or Age |__|__| years Sex Male Female

✂--

Anonymous code |__|__||__|__|__|__|

Clinical presentation Date of first symptoms |__|__| |__|__| |__|__|__|__|

Temperature |__|__|,|__| °C

**Headache**	No	Yes		**Myalgia**	No	Yes	
Prostration	No	Yes		Conjonctival suffusion	No	Yes	Unknown
Meningeal syndrome	No	Yes	Unknown	Anuria / Oliguria	No	Yes	Unknown
Proteinuria	No	Yes	Unknown	Icterus	No	Yes	Unknown
Haemorrages	No	Yes	Unknown	Cardiac arythmia	No	Yes	Unknown
Cardiac deficiency	No	Yes	Unknown	Skin rash	No	Yes	Unknown
Haemoptysis	No	Yes	Unknown	Others	No	Yes	Unknown

please state 1)____________ 2) ____________ 3) ____________

Antibiotic treatment since the first symptoms No Yes Unknown

If so, name of product ____________________ Date of first treatment |__|__| |__|__| |__|__|__|__|

Similar cases in entourage No Yes Unknown

Occupation(s) Occupation 1 ____________________ Occupation 2 ____________________

Freshwater exposition

Sewers, gutters	No	Yes	Unknown
River, creek	No	Yes	Unknown
Lake	No	Yes	Unknown
Swamps	No	Yes	Unknown
Irrigated culture	No	Yes	Unknown
Well	No	Yes	Unknown
Water tank	No	Yes	Unknown
Other	No	Yes	Unknown

*please state*____________________

Exposition context

Shower / Hygiene	No	Yes	Unknown
Swimming	No	Yes	Unknown
Fishing	No	Yes	Unknown
Hunting	No	Yes	Unknown
Cultures	No	Yes	Unknown
Building	No	Yes	Unknown
Military activities	No	Yes	Unknown
Other	No	Yes	Unknown

please state ____________________

Animal contacts during the 3 weeks before onset of illness

Dog / Cat	Non	Yes	Unknown	Sheep/Goat	Non	Yes	Unknown
Pork / Swine	Non	Yes	Unknown	Cattle	Non	Yes	Unknown
Horse	Non	Yes	Unknown				
Rodents	Non	Yes	Unknown	*Species* (rats / mice – Indoor / Outdoor)____________			
Other Mammals	Non	Yes	Unknown	*please state* ____________			

Suspected place of exposition (7-15 days before onset of illness): ____________________

Samples date sampled |__|__| |__|__| |__|__|__|__| please state Blood Urine

transmitted to IPNC on |__|__| |__|__| |__|__|__|__|

Disease outcome Recovery Recovery with sequela/state Death Unknown

Form completed by ____________________Tel / Email

Suspicion de leptospirose chez les patients hospitalisés

Critères d'inclusion : syndrome fébrile aigu avec céphalées et myalgies **ET AU MOINS UN des signes suivants** : prostration - injection conjonctivale – syndrome méningé – anurie ou oligurie et/ou protéinurie – ictère – hémorragies – arythmie cardiaque – insuffisance cardiaque – éruption cutanée

Lieu de recrutement Hôpital ______________________________ Service ____________________________________

Patient Date d'hospitalisation |__|__| |__|__| |__|__|__|__| Code anonymisation |__|__||__|__|__|__|

Nom ______________________________________ Prénom ____

Ville de résidence _____________________________________ Quartier _______________________________________

Point de repère du domicile ____

Date de naissance |__|__| |__|__| |__|__|__|__| Ou Age |__|__| ans Sexe Masculin Féminin

✂---

Code anonymisation |__|__||__|__|__|__|

Clinique Date des premiers symptômes |__|__| |__|__| |__|__|__|__|

Température axillaire corrigée (+0,5℃) ou rectale brute |__|__|,|__| ℃

**Céphalées**	Non	Oui		**Myalgies**	Non	Oui	
Prostration	Non	Oui		Injection conjonctivale	Non	Oui	Inconnu
Syndrome méningé	Non	Oui	Inconnu	Anurie/Oligurie	Non	Oui	Inconnu
Protéinurie	Non	Oui	Inconnu	Ictère	Non	Oui	Inconnu
Hémorragies	Non	Oui	Inconnu	Arythmie cardiaque	Non	Oui	Inconnu
Insuffisance cardiaque	Non	Oui	Inconnu	Eruption cutanée	Non	Oui	Inconnu
Hémoptysie	Non	Oui	Inconnu	Autres	Non	Oui	Inconnu

préciser 1)____________________________ 2) ____________________________ 3) ____________________________

Traitement antibiotique après le début de la maladie Non Oui Inconnu

Si oui, nom du produit __________________________________ Date de début du traitement |__|__| |__|__| |__|__|__|__|

Cas semblables dans l'entourage Non Oui Inconnu Si oui coordonnées ?

Activités professionnelles Activité 1________________________________ Activité 2________________________________

Contacts avec de l'eau douce

Caniveaux, égouts, fossés	Non	Oui	Inconnu
Rivière, ruisseau, canal	Non	Oui	Inconnu
Mare, étang, lac	Non	Oui	Inconnu
Marais	Non	Oui	Inconnu
Zone irriguée	Non	Oui	Inconnu
Puits	Non	Oui	Inconnu
Citerne, réservoir	Non	Oui	Inconnu
Autres	Non	Oui	Inconnu

*préciser*________________________________

Occasions de contact avec de l'eau douce

Toilette	Non	Oui	Inconnu
Baignade	Non	Oui	Inconnu
Pêche	Non	Oui	Inconnu
Chasse	Non	Oui	Inconnu
Activités agricoles	Non	Oui	Inconnu
Activités de construction	Non	Oui	Inconnu
Activités militaires	Non	Oui	Inconnu
Autres activités	Non	Oui	Inconnu

*préciser*________________________________

Contact avec des animaux dans les 3 semaines avant le début des signes

Chiens/chats	Non	Oui	Inconnu	Moutons/chèvres	Non	Oui	Inconnu
Porcs	Non	Oui	Inconnu	Bovins	Non	Oui	Inconnu
Chevaux	Non	Oui	Inconnu	Rats	Non	Oui	Inconnu
Autres rongeurs	Non	Oui	Inconnu	*préciser*____________________________			
Autres mammifères	Non	Oui	Inconnu	*préciser*____________________________			

Lieu de contamination suspecté (7 à 15 jours avant le début des symptômes) : ________________________________

Échantillons Prélevés le |__|__| |__|__| |__|__|__|__| Nature Sang Urine

Transmis à l'IPNC le |__|__| |__|__| |__|__|__|__|

Évolution de la maladie Guérison Guérison avec séquelles/Lesquelles Décès Inconnu

Fiche renseignée par ________________________________coordonnées tel/E-mail :
